# A short time interval between radiotherapy and hyperthermia reduces in-field recurrence and mortality in women with advanced cervical cancer

**DOI:** 10.1186/s13014-017-0813-0

**Published:** 2017-04-27

**Authors:** Caspar M. van Leeuwen, Arlene L. Oei, Kenneth W. T. K. Chin, Johannes Crezee, Arjan Bel, Anneke M. Westermann, Marrije R. Buist, Nicolaas A. P. Franken, Lukas J. A. Stalpers, H. Petra Kok

**Affiliations:** 1 Department of Radiation Oncology, Academic Medical Center, University of Amsterdam, Meibergdreef 9, 1105 AZ Amsterdam, The Netherlands; 2 Laboratory for Experimental Oncology and Radiobiology (LEXOR)/Center for Experimental Molecular Medicine, Academic Medical Center, University of Amsterdam, Meibergdreef 9, 1105 AZ Amsterdam, The Netherlands; 3 Department of Medical Oncology, Academic Medical Center, University of Amsterdam, Meibergdreef 9, 1105 AZ Amsterdam, The Netherlands; 4 Department of Obstetrics and Gynecology, Center for Gynecologic Oncology Amsterdam, Academic Medical Center, Meibergdreef 9, 1105 AZ Amsterdam, The Netherlands

**Keywords:** Radiotherapy, Hyperthermia, Time interval, Clinical outcome, Cervical cancer

## Abstract

**Background:**

Combined radiotherapy and hyperthermia is a well-established alternative to chemoradiotherapy for advanced stage cervical cancer patients with a contraindication for chemotherapy. Pre-clinical evidence suggests that the radiosensitizing effect of hyperthermia decreases substantially for time intervals between radiotherapy and hyperthermia as short as 1–2 h, but clinical evidence is limited. The purpose of this study is to determine the effect of the time interval between external beam radiotherapy (EBRT) and same-day hyperthermia on in-field recurrence rate, overall survival and late toxicity in women with advanced stage cervical cancer.

**Methods:**

Patients with advanced stage cervical cancer who underwent a full-course of curative daily EBRT and (4–5) weekly hyperthermia sessions between 1999 and 2014 were included for retrospective analysis. The mean time interval between EBRT fractions and same-day hyperthermia was calculated for each patient; the median thereof was used to divide the cohort in a ‘short’ and ‘long’ time-interval group. Kaplan-Meier analysis and stepwise Cox regression were used to compare the in-field recurrence and overall survival. Finally, high-grade (≥3) late toxicity was compared across time-interval groups. DNA repair suppression is an important hyperthermia mechanism, DNA damage repair kinetics were therefore studied in patient biopsies to support clinical findings.

**Results:**

Included were 58 patients. The 3-year in field recurrence rate was 18% and 53% in the short (≤79.2 min) and long (>79.2 min) time-interval group, respectively (*p* = 0.021); the 5-year overall survival was 52% and 17% respectively (*p* = 0.015). Differences between time-interval groups remained significant for both in-field recurrence (HR = 7.7, *p* = 0.007) and overall survival (HR = 2.3, *p* = 0.012) in multivariable Cox regression. No difference in toxicity was observed (*p* = 1.00), with only 6 and 5 events in the short and long group, respectively. The majority of DNA damage was repaired within 2 h, potentially explaining a reduced effectiveness of hyperthermia for long time intervals.

**Conclusions:**

A short time interval between EBRT and hyperthermia is associated with a lower risk of in-field recurrence and a better overall survival. There was no evidence for difference in late toxicity.

## Background

Cervical cancer is the fourth most common cancer in women worldwide, with 528,000 new cases and 266,000 deaths in 2012 [1]. Standard treatment for locally advanced cervical cancer is radiotherapy combined with weekly cisplatin-based chemotherapy [2]. Thermoradiotherapy, i.e. radiotherapy combined with hyperthermia, is a well-established alternative for patients with a contraindication for chemotherapy and provides similar overall survival [3–5].

Clinical thermoradiotherapy generally consists of fractionated daily external beam radiotherapy (EBRT) and, during the same period, weekly hyperthermia. In hyperthermia, the tumor is heated to a temperature of 40–43 °C for one hour. The rationale for adding hyperthermia to radiotherapy is that hyperthermia suppresses DNA double strand break (DSB) repair, the most lethal type of DNA damage caused by radiation treatment [6, 7]. Additionally, hyperthermia also sensitizes radioresistent (hypoxic) tumors by increasing oxygen delivery [8, 9]. This radiosensitizing effect increases the efficacy of the radiation treatment [10–13]. An EBRT fraction and hyperthermia session are usually given sequentially rather than simultaneously, since preclinical studies suggest that this results in the best therapeutic ratio [10, 14].

In clinical practice, the time interval between EBRT and hyperthermia treatment typically varies from 0.5–4 h for various reasons [5], such as availability of the treatment machines. Pre-clinical data suggest that longer time intervals between radiotherapy and hyperthermia reduce the radiosensitizing effect of thermoradiotherapy [14, 15], but clinical evidence is scarce. Only two studies investigated the effect of time interval, and only for a small and heterogeneous series of superficial tumors [16, 17].

Aim of this study is to determine the effect of the time interval between EBRT and hyperthermia treatments on in-field recurrence, overall survival and late toxicity in a retrospective cohort of cervical cancer patients. Furthermore, by examining the effect of time interval between EBRT and hyperthermia on the prevalence of DSBs in patient biopsies we study a potential mechanism supporting the clinically observed relationship.

## Methods

### Patient population

Included were patients treated at the Academic Medical Center for cervical cancer (ICD-9: 180, ICD-10: C53) with curative thermoradiotherapy, between January 1999 and January 2014. Excluded were patients who received concurrent chemotherapy and patients who received less than four out of the intended five hyperthermia sessions. Patients who received EBRT at other institutes were also excluded, because variation between institutes (e.g. different treatment guidelines, radiation schedules and techniques) would have introduced too many potential confounding factors.

All patients had a histologically confirmed cervical carcinoma, and were staged by FIGO clinical staging, including investigation under general anesthesia with cystoscopy, and lymph node staging by imaging (CT, MRI and/or PET). Patients with bulky lymph nodes (>2 cm) received a lymph node debulking first. Patients were referred for primary radiotherapy with hyperthermia for locally inoperable tumors (large FIGO IIB tumors, IIIA, IIIB and IVA) and for lymph node positive patients with FIGO IB and IIA. Since 2001, chemoradiotherapy became the standard treatment, and thermoradiotherapy was reserved for patients with a medical contraindication for cisplatin-based chemotherapy (i.e. hydronephrosis, renal insufficiency, poor performance).

In-field recurrence, overall survival and late toxicity data were extracted from patient files. Subsequently, overall survival for patients who were Dutch citizens was updated using the Dutch civil registry. Late toxicities, occurring or persisting at least 6 months after completion of thermoradiotherapy, were scored according to CTCAE v4.0. Only high-grade (≥3) toxicities were analysed, since retrospective analysis of low-grade toxicity is less reliable.

### Treatment

Treatment consisted of daily EBRT (23 x 2 Gy or 28 x 1.8 Gy) and five (occasionally four) weekly loco-regional hyperthermia treatments. At the end of the treatment period, a pulsed dose rate brachytherapy boost was given (24 Gy). Initially, EBRT was delivered using 3D conformal techniques, with a transition to IMRT in 2011. Hyperthermia was delivered during the period of EBRT treatment, approximately 1 h after the corresponding EBRT fraction. Hyperthermia was delivered by the AMC-4 phased array system, a 70 MHz radiofrequency heating system designed for deep-seated tumors [18]. A water bolus is attached to each antenna, to couple the electric field into the tissue and to cool the skin. An intravaginal E-field probe at the tumor location was used to determine the phase settings that yield optimal target heating. Temperatures were monitored during treatment using intracavitary 14-sensor thermocouple probes (spacing 0.5 cm, accuracy ±0.01 °C, ELLA, Czech Republic), placed in the vagina, bladder and rectum. Sensors located at positions indicative for the tumor were labelled as tumor in the monitoring software. Temperatures were measured every 30 s after a 5 s power off to avoid electromagnetic disturbance [19]. A steady state duration of 60 min was aimed for. Start of the steady state was defined as the moment when (after an initial warm-up period) one of the temperature sensors in the target region reached 41 °C, or 30 min after the start of the warm-up period if a temperature of 41 °C was not reached within that time.

### Statistical analysis

Since multiple hyperthermia treatments are delivered, treatment of a patient is not characterized by a single time interval between EBRT and hyperthermia. Thus, for each patient, the mean time interval (t_int,mean_) between their hyperthermia treatments and corresponding EBRT fractions was calculated. The median of t_int,mean_ was then used to split the population into a ‘short’ and a ‘long’ time-interval group.

The following patient and treatment characteristics were described for each time-interval group. Pre-treatment variables: age, histology, FIGO stage, lymph node status, smoking status. Hyperthermia parameters: tumor temperature (T_90,mean_), the steady state duration (HT duration_mean_), warm-up time, and number of hyperthermia treatments. T_90_ represents the temperature reached in at least 90% of the temperature measurement locations that are indicative for the tumor. T_90,mean_ is the mean T_90_ over each patient’s hyperthermia treatment series. Warm-up time was defined as the time between the start of power on to the start of the steady state. Differences between both time-interval groups in terms of these patient and treatment characteristics were tested for using Fisher’s exact test, the Chi-square test, the independent samples *t*-test and the Mann-Whitney *U* test depending on the type of data.

In-field recurrence rate and overall survival were calculated by the Kaplan-Meier method, and groups were compared by the log-rank test. Time to event (in-field recurrence or death) and censoring were calculated from the date of diagnosis. Multivariable analysis of in-field recurrence and overall survival was done by (backwards) stepwise Cox regression, including time-interval group, age, T_90,mean_, HT duration_mean_, histology, FIGO stage, lymph node status, number of hyperthermia treatments and smoking status as factors. A Fisher’s exact test was used to test for differences in the incidence of high-grade toxicity between time-interval groups. All analyses were performed using SPSS version 23, all tests were two-sided and *p* < 0.05 was considered significant. Accuracy of statistical estimates is reported using 95% confidence intervals.

### Patient biopsies

An effect of time interval on clinical outcome could be related to the amount of unrepaired DNA DSBs present at the time the hyperthermia is given, since DSB repair suppression is an important mechanism for the radiosensitizing effect of hyperthermia. To investigate this, experiments were performed on 12 cervical carcinoma biopsies. Biopsies were obtained from patients diagnosed in 2015 with advanced stage cervical cancer and eligible for thermoradiotherapy. Biopsies were divided in two parts: one half was treated *ex vivo* with radiotherapy (4 Gy), the other was left untreated (control). Six samples were fixated at approximately 15 min and six samples at approximately 2 h after radiotherapy. After treatment, biopsies were submerged in paraformaldehyde, to be used for paraffin coupes. Before antigen retrieval, they were deparaffinized and rehydrated. Afterwards a heat-induced antigen retrieval at pH 9.0 for 20 min was performed, followed by a 30 min cooling period. Next, a 15 min PO block including H_2_O_2_ was performed. Then coupes were incubated overnight at 4 °C with γ-H2AX mAb (Millipore, Merck). Next, tissue was embedded in Alexa Fluor 488 (Invitrogen Life Technologies), after washing with PBS. DAPI was used to stain the nuclei blue before covering tissue with a drop of ProLong Gold anti-fade reagent (Invitrogen Life Technologies) and a coverslip.

## Results

Fifty-eight patients were included. The median time interval was 79.2 min, defining the short and long time-interval groups as t_int,mean_ ≤ 79.2 min (33.8–79.2 min) and t_int,mean_ > 79.2 min (80.0–125.2 min) respectively. Out of all clinical and treatment characteristics, only warm-up time and the time interval itself were significantly different across time-interval groups (Table 1). Median follow-up for censored patients was 18 months (range, 2–130 months) for in-field recurrence and 37 months (range, 3–195 months) for overall survival.

**Table 1:** Characteristics of the included patients, stratified by the mean time interval between radiotherapy and hyperthermia

	Short group (*n* = 30)		Long group (*n* = 28)		Statistical test	*p*
	Median (range)		Median (range)			
t_int,mean_ [min]	65.8 (33.8–79.2)		91.7 (80.0–125.2)		Mann-Whitney *U* test	<0.001
Age [y]	67.5 (33–90)		65.0 (29–85)		Mann-Whitney *U* test	0.45
T_90,mean_ [°C]	40.0 (38.6–41.9)		40.3 (38.2–41.1)		*T*-test	0.71
HT duration_mean_ [min]	60.0 (52.6–63.8)		60.8 (34.5–64.6)		Mann-Whitney *U* test	0.16
Warm-up time [min]	5.2 (0.75–17.8)		8.5 (2.75–26.6)		Mann-Whitney *U* test	0.001
	N	%	N	%		
Histology					Fisher’s exact test	1.00
Squamous cell carcinoma	27	90	26	93		
Adenocarcinoma	3	10	2	7		
Figo stage					Chi-square test	0.40
IB	3	10	4	14		
IIA	1	3	0	0		
IIB	6	20	8	29		
IIIA	2	7	5	18		
IIIB	14	47	10	36		
IVA	4	13	1	4		
Lymph node status					Fisher’s exact test	1.00
Negative	15	50	14	50		
Positive	15	50	14	50		
Number of hyperthermia treatments					Fisher’s exact test	0.22
4	5	17	9	32		
5	25	83	19	68		
Smoking					Fisher’s exact test	1.00
Yes	7	23	7	25		
No	23	77	21	75		

**Fig 1:**
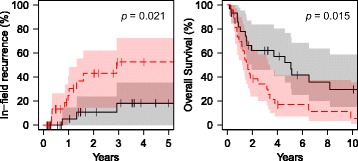
Kaplan-Meier survival analysis for in-field recurrence (left) and overall survival (right) for the short time-interval group (*black*) and the long time-interval group (*red*). Shaded area represents the 95% confidence interval

The in-field recurrence rate and overall survival were significantly better in the short time interval group (Fig. 1). The 3-year in-field recurrence rate was 18% (0–35%) in the short time-interval group and 53% (18–82%) in the long time-interval group. The 5-year overall survival was 52% (35–77%) in the short time-interval group and 17% (7–41%) in the long time-interval group; median overall survival was 61 months (38–83 months) and 19 months (13–26 months) respectively.

The last iteration of the stepwise Cox regression for in-field recurrence included three significant factors for a favorable outcome: a short time interval, advanced age and long warm-up time (Table 2). In the overall survival analysis, the last iteration included three prognostically favorable factors: a short time interval (significant), high T_90,mean_ (significant) and negative lymph node status (trend, not significant).

**Table 2:** Last iterations of the backwards stepwise Cox regression for in-field recurrence and overall survival

In-field recurrence	Factor	HR (95%CI)	*p*
	Time-interval group	7.7 (1.8–33.8)	0.007
	Age	0.96 (0.92–1.00)	0.048
	Warm-up time	0.85 (0.72–1.00)	0.047
Overall survival	Time-interval group	2.3 (1.2–4.5)	0.012
	T_90,mean_	0.57 (0.39–0.87)	0.009
	Nodal status	1.8 (0.91–3.4)	0.093

Six high-grade late toxicities were observed in the short time-interval group, compared to five high-grade toxicities in the long time-interval group (Table 3, *p* = 1.00).

**Table 3:** Number and type of late toxicities, stratified by time-interval group

CTC-score	Short group	Long group
<3	Unspecified (16)	Unspecified (17)
3	Radiation cystitis (1), Pelvic fracture (1), rectovaginal fistula (1), local radiation ulcer (1)	Local radiation ulcer (1)
4	Secondary in-field malignancy (1), local radiation ulcer (1)	Radiation enteritis (1), complex/multiple (1), vesicovaginal fistula (1)
5	-	Gastrointestinal perforation (1)
Insufficient follow-up	8	6

**Fig 2:**
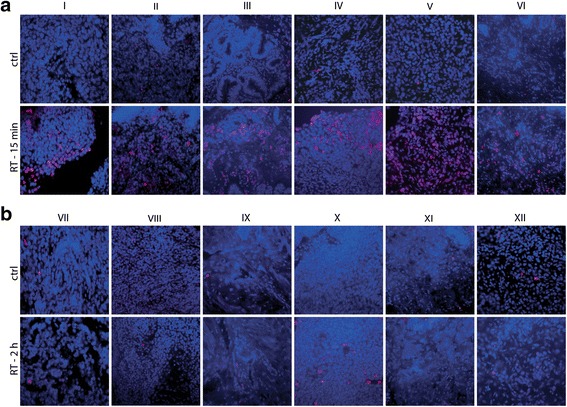
γ-H2AX staining showing unrepaired DNA double strand breaks (*in red*) in patient biopsies at 15 min (**a**) and 2 h (**b**) after 4 Gy irradiation (RT) compared to control (ctrl). Roman numerals identify individual biopsies

The γ-H2AX staining of patient biopsies showed a substantial increase in DSBs for the six samples fixated 15 min after irradiation, compared to control (Fig. 2a). For the six samples fixated at two hours, the number of DSBs was similar to that of untreated samples (Fig. 2b). Patients included in this retrospective study had time intervals between EBRT and hyperthermia ranging from 30 min to two hours. Thus, patients with short time intervals received hyperthermia when substantial DNA damage was still present, while patients with long time intervals received hyperthermia when the majority of DNA damage was already repaired.

## Discussion

This is the first clinical study to demonstrate the effect of time interval on treatment outcome in patients with advanced stage cervical cancer. The results of both univariable and multivariable analyses indicate that a short time interval between EBRT and hyperthermia treatments results in a lower in-field recurrence rate and better overall survival. Although the confidence intervals were substantial due to the limited size of the patient group, the estimated effect size was large enough to yield a significant result. At the same time, the results provide no evidence for an effect of time interval on high-grade late toxicity; a possible relationship may however have been obscured by the low number of toxicity events.

In addition to time-interval group, a significant correlation between age and in-field recurrence rate was identified in the multivariable analysis, with older patients having less recurrences (Table 2). This effect may be explained by our patient policy. While elderly women were often denied chemotherapy due to their generally frail condition, younger patients usually received thermoradiotherapy instead of chemoradiotherapy because of hydronephrosis. This results in a selection of young patients with relatively large local tumors and/or extensive lymph node metastasis. Warm-up time was also identified as a significant factor, where patients with longer warm-up time did better. After warm-up, a fixed duration of steady state (60 min) is aimed for. Thus, patients with longer warm-up time will have a longer total heating time, and have therefore received a higher thermal dose. Comparing the patients in our study with the shortest and longest warmup times (approximately 1-25 min), the difference in CEM43 was about 10–15%. The observed significance of warm-up time can therefore be understood in view of the well-established correlation between thermal dose and clinical outcome [20–25]. However, the estimated hazard ratio (0.85 min^-1^) is uncertain (i.e. has a large 95% confidence interval) and appears to be relatively low considering the modest difference in CEM43. For overall survival, a high T_90,mean_ was identified as a significantly favorable factor, also supporting a thermal dose effect relationship.

Biological studies in the late 70’s already suggested that the time interval between irradiation and hyperthermia affects outcome [14, 15]. These in vitro and in vivo experiments have shown that the radiosensitizing effect of hyperthermia in tumor tissue decays substantially in the first 2 h, in particular when EBRT is given before hyperthermia (Fig. 2 in Li et al [15] and Fig. 5 in Overgaard [14]). These pre-clinical experiments were performed at higher temperatures (42.5–43 °C) than what is generally achieved in the clinic, and the contribution of the various mechanisms of hyperthermia (e.g. DNA repair inhibition, reoxygenation and direct cytotoxicity) may be different at lower temperatures [26]. Nonetheless, these data corroborate the difference in local control observed in this study. Additionally, Overgaard’s data suggest that the radiosensitizing effect decays even more rapidly in normal tissue, regardless of the order in which radiotherapy and hyperthermia are applied. If a substantial part of the radiosensitizing effect in normal tissue has already disappeared for a time interval of one hour, this could explain why no difference in late toxicity was observed between the short time-interval group (median 65.8 min) and the long time-interval group (median 91.7 min).

Prior to this study, clinical evidence on the effect of time interval was limited to the results of two small studies, which both investigated superficial recurrent and metastatic tumors of mixed primary origin. Lindholm et al compared time intervals of 0.5–1.5 h to 3–4 h and saw no significant difference between both groups in terms of either tumor response or skin toxicity [16]. However, the short time-interval group in this study only included 15 tumors and time-interval groups were not comparable with respect to the numbers and mode of hyperthermia treatment. Arcangeli et al described three trials involving hyperthermia [17]. The second trial compared hyperthermia immediately after radiotherapy, with delayed hyperthermia (4 h between treatments). Reported local tumor control at 6 months was 5/7 for immediate hyperthermia compared to 4/7 for delayed hyperthermia. Moist desquamation occurred in 64% and 46% of the cases for immediate and delayed hyperthermia respectively. However, definite conclusions could not be drawn because of the small numbers. Additionally, the results of both studies may not be applicable to modern hyperthermia, because of substantial improvements in hyperthermia treatment techniques resulting in better treatment quality.

In a more recent study, a more homogenous group of superficial tumors (all recurrent breast cancers) was studied [27]. Hyperthermia was applied after radiation and one factor that was investigated was whether patients received EBRT and hyperthermia in the same or in different institutes. For patients treated within a single institute, local control was worse (not significant) and late toxicity was increased (significant). However, treatment outcomes generally vary between institutes due to e.g. differences in patient selection and treatment. Thus, the actual effect of time interval is very difficult to determine from these results, since any relation between the factor ‘institute’ and treatment outcome cannot be exclusively attributed to an effect of time interval.

Multiple mechanisms have been suggested for the radiosensitizing effect of hyperthermia [26]. In our institute, hyperthermia is delivered after radiation, and the ability of heat to interfere with DNA damage repair is an important mechanism for radiosensitization for this treatment sequence [6, 7]. This mechanism can only be effective if unrepaired DNA damage is still present. Radiobiological studies on clinical data have shown that the exponential repair time constant for radiation damage is roughly 1.5 h [28–31]. A similar repair time is suggested by the results of our experiments on patient biopsies. While a substantial amount of DNA DSBs was observed 15 min after irradiation (Fig. 2a), almost all DNA damage was repaired after two hours (Fig. 2b). As time intervals in the long time-interval group ranged up to 2 h, our biopsy data could explain why hyperthermia is less effective in this group: since much of the radiation damage has already been repaired when hyperthermia is given, the efficacy of the repair-blocking mechanism is substantially reduced. In contrast, a substantial amount of unrepaired damage is still present at the time of hyperthermia treatment in the short time-interval group, thus the repair-blocking mechanism is effective. It is important to note that when radiation is delivered after heat, increased perfusion and corresponding reoxygenation may become more important mechanisms for radiosensitization than DNA repair inhibition [32]. Thus, for this order of treatments, the dynamics of radiosensitization may be different from what was observed in this study and additional research is needed to determine the effect of time interval for hyperthermia applied before radiation.

The Dutch Deep Hyperthermia Trial (DDHT), which compared radiotherapy to thermoradiotherapy in advanced stage cervical cancer, reported a 3-year overall survival of 27% for the radiotherapy group and 51% for the thermoradiotherapy group [33]. In our study, the 3-year overall survival for all 58 patients was 48% (36–64%), almost similar as in the DDHT. However, 3-year overall survival was considerably lower in the long time-interval group at 34% (20–59%) compared to 62% (46–84%) in the short time-interval group. Comparison of the two series could lead to two conclusions. First, the overall survival in the long time-interval group in this study was close to that of the radiotherapy-alone arm in the DDHT trial, suggesting that long time-interval patients had very little benefit from the hyperthermia treatment. Second, if a short time interval can be ensured for all patients, an additional improvement of approximately 10% in overall survival of the results in the DDHT may be attained.

Our findings may have important consequences for treatment policy of women with inoperable cervical cancer. Although underpowered, results from a trial comparing standard chemoradiotherapy with thermoradiotherapy in women with inoperable cervical cancer suggest that chemoradiotherapy and thermoradiotherapy are equally effective, with approximately 60% long term event free survival in both arms [5]. However, time interval between EBRT and hyperthermia in this trial ranged from 1–4 h, which may have resulted in sub-optimal results for the thermoradiotherapy group. Thus, thermoradiotherapy could potentially be even more effective than standard chemoradiotherapy, provided a short time interval between EBRT and hyperthermia is ensured. This hypothesis would need confirmation through a clinical trial, and would require EBRT and hyperthermia to be delivered with a short time interval.

Recently, a systematic review showed the potential value of thermochemoradiotherapy in the treatment of locally advanced cervical cancer, but concluded that further confirmation through prospective randomized trials is needed [34]. This raises the question whether the effect of time interval between radiotherapy and hyperthermia is equally important in thermochemoradiotherapy, in which case imposing strict limits to the time interval should be considered in designing such a trial. Hyperthermia and cisplatin primarily inhibit different DNA repair pathways (homologues recombination and non-homologous end-joining, respectively) [7, 35, 36]. Although blocking additional repair pathways may cause activation or a shift to other pathways (potentially causing a change in dynamics), it seems unlikely that the dynamics of the interaction between hyperthermia and radiotherapy would be changed dramatically. While no definitive proof is available, limiting the time interval seems a reasonable approach when aiming at an optimal synergistic action of the three modalities in a thermochemoradiotherapy trial.

The importance of a short time interval has implications for clinical practice. In recent years an increasing number of patients received hyperthermia at our institute, but radiation treatment elsewhere (5 out of 11 patients in 2014). This is more convenient for patients, since the hyperthermia center (generally a longer commute than the nearest radiotherapy center) then only needs to be visited once a week. However, considering the substantial reduction in efficacy of the hyperthermia, delivering both treatments in separate institutes should be strongly discouraged. A solution would be for patients to receive EBRT on the day of hyperthermia within the same institute, while all other EBRT treatments are delivered in a center closer to the patients residence. However, this requires radiotherapy treatment plans to be designed for both institutes and will yield additional workload. Even when both treatments are given within a single institute, long time intervals should be avoided, and this is now standard practice for locoregional hyperthermia at our institute. While the optimal time interval cannot be established based on the current data and an increase in normal toxicity may be expected for very short time intervals based on pre-clinical data, a time interval of 1 h appears to be a reasonable tradeoff between feasibility, efficacy and safety [14, 15].

## Conclusion

A short time interval between EBRT and hyperthermia is associated with a lower risk of in-field recurrence and a better overall survival. Efficacy is reduced for longer time intervals, likely because of the reduced amount of unrepaired DNA damage present at the time of hyperthermia treatment. There was no evidence for a difference in long-term toxicity, however, the low number of events in both arms means that statistical power is limited. Limiting the time interval between EBRT and hyperthermia to approximately one hour is recommended.

## Abbreviations

DDHT: Dutch deep hyperthermia trial
DSB: double strand break
EBRT: external beam radiotherapy
